# Cardiac Computed Tomography: Technological Developments and Clinical Applications

**DOI:** 10.3390/jcdd12120473

**Published:** 2025-12-02

**Authors:** Katsuya Suzuki, Hiroyuki Takaoka, Ryosuke Irie, Moe Matsumoto, Yoshitada Noguchi, Shuhei Aoki, Kazuki Yoshida, Haruto Matsumoto, Satomi Yashima, Makiko Kinoshita, Haruka Sasaki, Noriko Suzuki-Eguchi, Yoshio Kobayashi

**Affiliations:** 1 Department of Cardiovascular Medicine, Chiba University Graduate School of Medicine, Chiba 260-0856, Japan; 2 Department of Radiology, Chiba University Hospital, Chiba 260-8677, Japan

**Keywords:** cardiac computed tomography, coronary artery disease, myocardial disease

## Abstract

Cardiac computed tomography (CT) has long evolved as a highly accurate screening tool for coronary artery disease. New technologies such as multi-detector rows and artifact reduction by a new motion correction algorithm have made it possible to evaluate coronary artery stenosis with higher diagnostic accuracy and lower radiation exposure. In addition to the anatomical evaluation of coronary arteries, the introduction of fluid dynamic analysis enables the measurement of coronary fractional flow reserve (FFR) for each stenotic lesion, which can only be achieved through invasive catheter evaluation. Myocardial ischemia can now also be detected using myocardial stress perfusion CT imaging. In addition, with the advent of dual-energy imaging or new image reconstruction technology, the addition of late contrast phase imaging enables myocardial late enhancement and left ventricular (LV) extracellular volume (ECV) analysis, which was previously possible only with cardiac magnetic resonance imaging (MRI). It has also been reported that LV ECV may be useful in predicting prognosis in cases with cardiomyopathies. In addition, retrospective imaging of the entire heart in a single cardiac cycle is now possible with lower radiation exposure, enabling not only morphological evaluation of the heart and valves but also myocardial strain analysis, which has conventionally been evaluated mainly by echocardiography and is expected to be applied in clinical practice in the future. Cardiac CT, which overcomes the weaknesses of other modalities while demonstrating greater usefulness through the latest technological development, is expected to expand its field of application to the entire heart analysis. The purpose of this review is to provide an overview of the technological development of cardiac CT, which has seen remarkable development in recent years, along with its clinical utility, with the aim of enabling clinicians to fully utilize it in daily practice.

## 1. Introduction

Cardiac computed tomography (CT) has recently become widely used to screen for coronary artery disease in cardiovascular practice [1]. The advent of multi-row detectors, high speed gantry rotation, new image reconstruction methods, and motion correction algorithms (MCA) has enabled the stable detection of anatomical coronary artery stenosis with high diagnostic accuracy at low radiation doses, even in arrhythmia cases such as atrial fibrillation. Furthermore, the emergence of fractional flow reserve CT (FFR-CT) has made it possible to detect functionally significant coronary artery stenosis as well (Table 1).

Beyond this application, advances in CT technology have made it possible to detect left ventricular (LV) myocardial fibrosis with late phase contrast imaging and to analyze cardiac function over the cardiac cycle with reduced radiation exposure [2]. In addition, 4-dimensional imaging allows aortic valve motion analysis. Current guidelines also recommend the use of CT for valvular disease severity assessment, especially for calcification scoring with non-contrast imaging [3]. Here, we present innovations in cardiac CT and their clinical applications, with some reports (Table 1).

## 2. Evaluation of Coronary Artery Stenosis

With appropriate patient selection, coronary CT angiography (CTA) is highly accurate in detecting significant coronary artery stenosis, particularly because of its high negative predictive value [4]. It is of interest as a useful diagnostic and prognostic imaging test in low- to intermediate-risk patients with symptoms consistent with angina pectoris and no history of coronary artery disease [5].

### 2.1. Motion Correction Algorithm on Cardiac Computed Tomography

**Figure 1:**
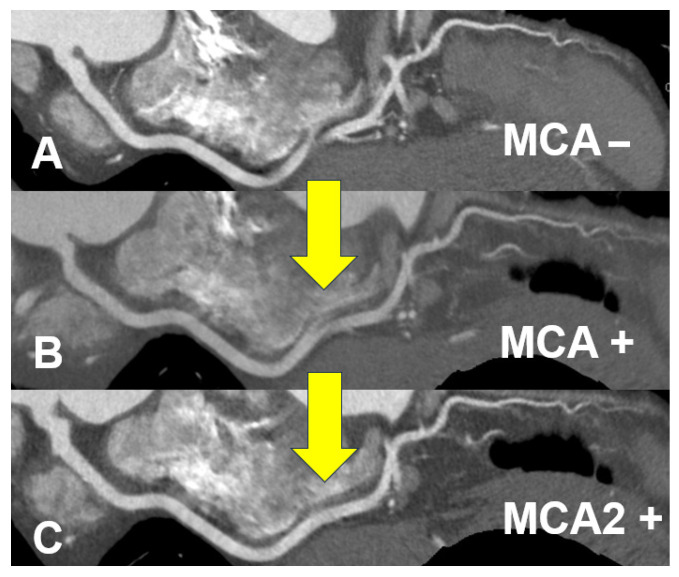
Improvement of motion artifacts in the right coronary artery using a novel motion correction algorithm. From top to bottom: image without motion correction algorithm (MCA) applied (**A**), image with first-generation MCA applied (**B**), image with second-generation MCA applied (**C**). Motion artifacts are progressively reduced by the novel motion correction algorithm (yellow arrows).

The advent of new MCA has improved the diagnostic accuracy of significant coronary artery stenosis, especially in patients with high heart rates [6]. First, we will explain GE HealthCare’s Motion Correction Algorithm, SnapShot Freeze (SSF) (2). After completing multi-phase cardiac reconstruction and automated coronary vessel tracking, SSF integrates temporal data from neighboring cardiac phases within a single heartbeat to model the trajectory and speed of vessel motion. Using this information, the algorithm estimates the vessel’s true spatial position at the designated target phase (Figure 1). This adaptive process compensates for residual motion artifacts and effectively reduces the temporal width of the reconstruction window. Motion correction is applied individually to each vessel and segment, accounting for phase-dependent variations in voxel motion across the coronary tree [7]. The second-generation motion correction algorithm has been newly introduced, allowing for the acquisition of higher-precision images of the coronary arteries (Figure 1). Building on the use of conjugate pairs of partial-angle reconstruction images, SSF2 automatically estimates and compensates for cardiac motion throughout the image volume. For each region, the algorithm identifies a local motion trajectory that aligns with the subset of projection data intersecting that area. Once the trajectory is established, the raw data are divided into temporal subsets based on the acquisition time of the corresponding projection rays. Each reconstructed volume is then spatially transformed according to the motion field that maps its temporal state to the central reference phase. Because whole-heart correction requires motion estimation in all three spatial directions, this approach enhances the robustness of coronary motion correction, particularly in cases of pronounced motion or dominant *z*-axis displacement [7]. Such motion correction is also possible with devices from other vendors, and reports on its usefulness have been emerging [8]. Additionally, it reduces motion artifacts in other structures, such as the aortic valve. This improvement is particularly beneficial for analyses like assessing the aortic valve before transcatheter aortic valve implantation (TAVI).

### 2.2. Plaque Analysis of Coronary Artery on CT

The advantages of CT include not only its minimally invasive nature and ability to assess the presence of severe coronary artery stenosis, but also its simultaneous capability to evaluate plaque characteristics within stenotic lesions. This coronary plaque characterization is known to be useful for predicting the future risk of acute coronary syndrome following CT. Specifically, characteristics indicative of unstable plaque include low CT values, spotty calcification, and positive remodeling [9]. Furthermore, in the SCOT-HEART trial, one reason cardiac CT-guided initial patient management proved more effective than standard care in improving outcomes was the higher rate of initiation of optimal medical therapy (OMT) for secondary prevention compared to the standard care group. It is anticipated that enhanced risk management based on such coronary plaque assessment will lead to improved patient outcomes [10].

Recent large-scale studies have demonstrated that CCTA techniques visualizing and quantifying not only the “burden (volume)” but also the “quality (composition and vulnerability)” of coronary plaque are highly powerful in predicting prognosis. Specifically, a subanalysis of the SCOT-HEART trial found that low-attenuation plaque (LAP) volume was one of the strongest predictors of fatal and non-fatal myocardial infarction [11].

Furthermore, the long-term follow-up study of the PARADIGM registry reported that serial CCTA closely monitored numerous patients with mild coronary artery disease (25–49% stenosis), revealing that statin therapy not only suppressed plaque progression but also induced qualitative changes in plaque (reduction in necrotic cores, progression of calcification) [12].

Additionally, PARADIGM data enabled the development of a prediction score for non-obstructive CAD patients (incorporating spot calcification, low-density components, stenosis percentage, bifurcation plaque count, etc.), which is expected to guide indications for follow-up CCTA and serve as an indicator for intensified treatment strategies [13].

Meanwhile, large observational data such as the CONFIRM registry also show that proximal plaque burden (especially mixed/calcified plaque) is an independent predictor of all-cause mortality. Information on plaque composition and location obtained from CCTA adds prognostic predictive value beyond clinical risk factors and calcium scores [14].

Recently, systematic reviews/meta-analyses have also confirmed that quantitative plaque volume measured by CCTA is a powerful biomarker predicting major cardiovascular events. Furthermore, research on deep learning-based plaque quantification is advancing. Methods using deep learning to rapidly and reproducibly assess total plaque volume have been reported as independent predictors of myocardial infarction. Collectively, these findings indicate that CCTA plaque assessment is establishing itself as a highly powerful tool beyond mere anatomical stenosis diagnosis, particularly in terms of future risk prediction, treatment monitoring, and designing rescan strategies [15].

### 2.3. Innovation in New Scanner Technology of Computed Tomography

When cardiac CT was first introduced, its high radiation exposure levels were a significant concern. Reducing radiation exposure during cardiac CT is particularly important for younger patients, and recent advances in scanner technology have significantly reduced exposure levels during cardiac CT examinations [16].

First, the introduction of longitudinal wide-coverage multislice detector rows has been one of the most impactful innovations. While this development is notable for reducing coronary stepping artifacts in arrhythmia cases, it also eliminates overlapping scan ranges, which plays an essential role in reducing radiation exposure [16].

**Figure 2:**
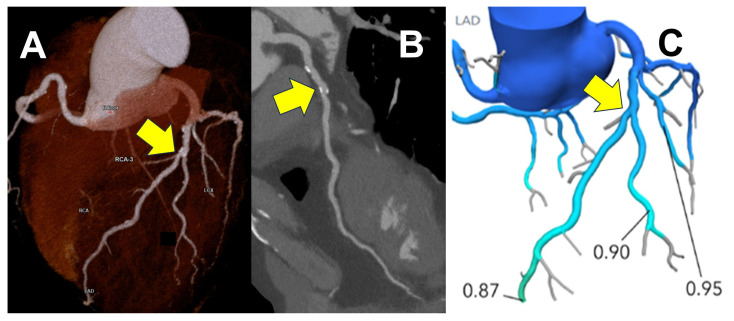
FFR-CT enables detailed noninvasive coronary artery assessment in cases where invasive testing is undesirable. This is a coronary CT image of a patient before bentall procedure. Findings suggestive of moderate stenosis were suspected in the left anterior descending artery (LAD), necessitating a more detailed preoperative ischemic evaluation (**A**,**B**) (yellow arrows). Since invasive coronary angiography was risky, FFR-CT was performed (**C**). It revealed no functional ischemia in the coronary arteries, including the LAD (yellow arrow), allowing avoidance of invasive testing.

Furthermore, new image reconstruction techniques such as iterative reconstruction and deep learning reconstruction (DLR) have enabled the acquisition of high-quality images with low noise even at low radiation doses, compared to conventional filtered back projection (FBP). These techniques have been demonstrated to be effective in significantly reducing radiation exposure while maintaining high image quality in coronary angiography [17]. Such noise reduction using advanced reconstruction techniques is also beneficial for myocardial evaluation (Figure 2). 

Photon-counting computed tomography (PCCT) represents a transformative advance in CT technology. Unlike conventional energy-integrating detectors, this new generation of scanners employs energy-resolving detectors capable of counting individual x-ray photons and determining their energy levels. By directly measuring photon interactions, photon-counting CT achieves superior contrast-to-noise ratios, enhanced spatial resolution, and more efficient spectral imaging capabilities [18]. These improvements translate into several clinical advantages: lower radiation doses, high-resolution image reconstruction, reduction in beam-hardening artifacts, more efficient use of contrast material, and expanded opportunities for quantitative tissue characterization [18]. Si-Mohamed SA et al. reported the results of the first application of PCCT to actual 14 cases, demonstrating that it reduced radiation exposure compared to conventional, while improving image quality for calcified and non-calcified plaques, as well as stent lesions [19]. Araki S et al. demonstrated that high-pitch +70 kVp scanning using photon-counting CT maintained image quality and the reliability of stenosis assessment while limiting the effective dose to an average of 0.41 mSv [20]. Zou LM et al. demonstrated that CCTA using super-resolution deep learning reconstruction (SR-DLR) reduced radiation dose by 60% (2.01 → 0.80 mSv) without compromising plaque quantification, composition, or stenosis assessment [21].

Ultra High Resolution (UHR)-CT, which offers higher spatial resolution than conventional systems, has also emerged, and its usefulness for coronary artery assessment is generating discussion. Motoyama S et al. previously reported UHR-CT’s high spatial resolution reduces blooming artifacts in calcified plaques and improves the accuracy of stenosis assessment [22]. It provides superior visualization of the stent lumen compared to conventional CT, enabling more quantitative and reliable assessment of lumen diameter and strut evaluation. UHR-CT also excels in quantifying plaque characteristics (fibrous plaque, etc.), vascular wall volume, and wall-to-lumen ratio, allowing for more detailed assessment than conventional resolution [23].

### 2.4. Fractional Flow Reserve Analysis Using Computed Tomography

**Figure 3:**
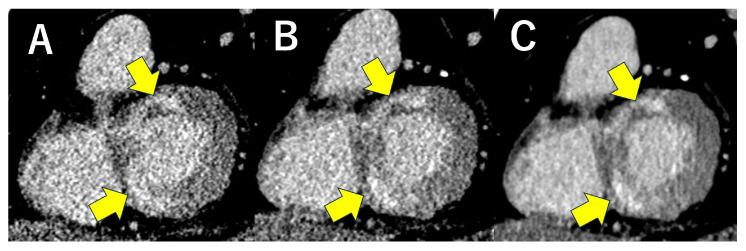
Advanced reconstruction techniques for noise reduction are also beneficial in myocardial assessment. These are delayed-phase images from the same case with cardiac sarcoidosis. As reconstruction techniques improved (Filtered back projection (**A**) → Hybrid image reconstruction (**B**) → Deep learning reconstruction (**C**)), image noise was reduced, and the contrast of delayed enhancement became more distinct (yellow arrows).

Computational Fluid Dynamics (CFD) applied to coronary CTA images is a new technique that allows the prediction of coronary artery blood flow and intracoronary pressure, and the calculation of lesion-specific coronary blood fractional flow reserve (FFR) [24]. FFR can be calculated from usually taken coronary CTA scans (FFR-CT) and has been reported to improve the diagnostic accuracy of coronary lesions compared to visual analysis of coronary CT alone and to correlate well with invasive FFR [25]. Norgaard BL et al. reported in the NXT trial that when comparing coronary CTA and FFR-CT using invasive FFR-detected ischemia as the standard, the analysis of 254 cases demonstrated improved sensitivity and positive predictive value for moderate stenotic lesions [26]. Combined with a physiological assessment of coronary lesions, this approach can improve event-free survival, reduce unnecessary invasive testing, lower healthcare costs, and enhance clinical decision-making (Figure 3). The ADVANCE registry is a trial that examined how treatment decisions determined by coronary CTA changed when FFR-CT was added. Within this registry, Fairbairn, T. A. et al. reported that while 53% of cases were deemed to require additional testing based on coronary CTA evaluation at the core lab, none of these cases were ultimately judged to require additional testing after adding FFR-CT. In practice, medical therapy was performed in 75% of cases. Overall, adding FFR-CT led to a change in treatment plan in 66.9% of cases. Furthermore, cases with an FFR value of 0.8 or higher by FFR-CT had zero cardiovascular events at 90 days, whereas 0.6% of cases with an FFR value below 0.8 experienced a major cardiovascular event [27]. The PLATFORM trial is a randomized study comparing FFR-CT assessment with conventional methods in two groups: one scheduled for noninvasive testing and the other for invasive testing in the diagnosis of coronary artery disease. Within this trial, Douglas et al. reported that performing FFR-CT prior to invasive testing eliminated the need for invasive coronary angiography in 61% of cases [28]. Furthermore, the introduction of FFR-CT did not increase cardiovascular events at the one-year follow-up [29]. Recent studies have reported that the new fluid dynamic analysis algorithm is useful for assessing functionally significant lesions [30].

Furthermore, in the DYNAMIC-FFRCT registry, approximately 40% of patients scheduled for coronary angiography avoided the procedure due to FFR-CT results. Additionally, cases with FFR > 0.80 required minimal additional testing, confirming potential cost savings [31]. The IMPACT-FFR study applied FFR-CT to over 2000 CCTA cases, conducting a comprehensive analysis of clinical pathways, prognosis, and healthcare economics. One-year mortality and non-fatal myocardial infarction rates remained low, demonstrating the safety of cohort management. However, the study clarified that the introduction cost poses a challenge from a healthcare economics perspective, and cost-effectiveness depends on the regional healthcare system [32].

The TARGET trial showed that introducing on-site CT FFR analysis reduced unnecessary invasive coronary angiography (ICA) and optimized diagnostic flow. However, it did not demonstrate a reduction in major adverse cardiac events (MACE) within 90 days or an improvement in quality of life (QOL) [33]. The post-stent FFRCT prognostic study applied machine learning-based CT FFR analysis to stent placement cases, reporting that the difference in FFR between proximal and distal segments on CT and stent length are independent predictors of long-term MACE. This demonstrated the role of CT FFR analysis as a noninvasive stent follow-up method [34]

## 3. Technological Improvement of Computed Tomography for Assessment of Myocardial Damage

### 3.1. Dual Energy/Photon Counting

Dual Energy CT is an imaging technique that utilizes the phenomenon where the linear attenuation coefficient of substances changes by employing different X-ray energies. This technology provides information such as virtual monochromatic X-ray images and substance discrimination images [35]. Ohta Y et al. reported the utility of DECT to detect late enhancement of left ventricular myocardium in 44 patients with heart failure, and they reported that its sensitivity and specificity to detect late enhancement were 92% and 98% compared to the gold standard MRI in the segment-based analysis [36].

Tremamunno G et al. also reported results from applying PCCT to evaluate myocardial late enhancement in 27 cases, and they revealed over 90% diagnostic accuracy to detect segmental late enhancement on PCCT, compared with MRI [37].

### 3.2. Late Enhancement and Extracellular Volume Analysis on Computed Tomography

These advances in high-definition image acquisition techniques have recently enabled cardiac CT to detect myocardial fibrosis as late enhancement by adding a late contrast phase scan a few minutes after the early phase of coronary CT angiography, which has been used in some clinical studies [38]. Although this delayed contrast effect was traditionally assessed using magnetic resonance imaging (MRI), recent advances in cardiac CT—especially reduced radiation dose and improved image quality in the late phase scan—have made its use in routine clinical practice more feasible. With modern equipment, the ability of CT to detect LV late enhancement is now considered comparable to MRI [39].

**Figure 4:**
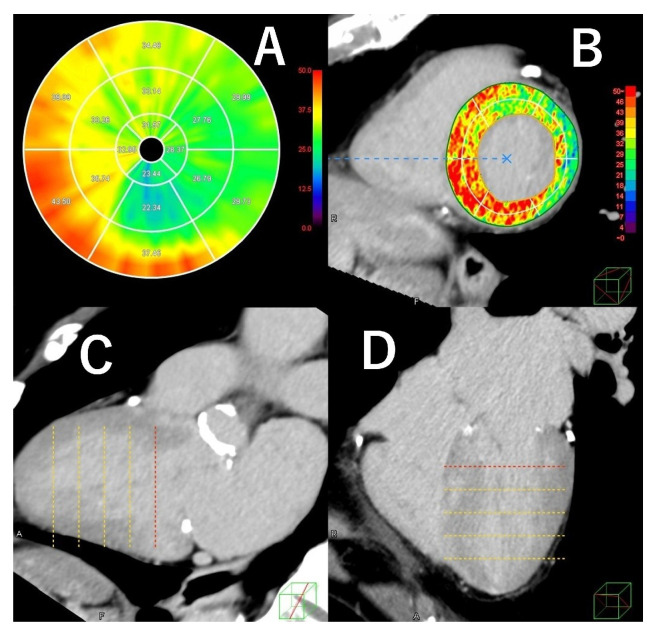
This is a case of severe aortic stenosis in which ECV analysis of the left ventricular myocardium (LVM) was performed using pre-TAVI CT (**A**). The mean ECV of the LVM was 33.6%. This patient was diagnosed with cardiac amyloidosis two years after TAVI. The short-axis view, two-chamber view, and four-chamber view of delayed contrast LVM are shown in Figures (**B**–**D**).

Assessment of LV late enhancement was originally useful in differentiating underlying myocardial disease based on enhancement patterns, and modern CT scanners have also proven effective in this differentiation [40]. Recently, the development of new image analysis software, such as quantifying left ventricular extracellular volume (LV-ECV), has enabled qualitative and quantitative assessment of late enhancement (Figure 4).

The appearance of T1 mapping in MRI has enabled the assessment of LV-ECV [41]. Based on biopsy findings, it correlates well with the histopathological burden of myocardial fibrosis in dilated cardiomyopathy and is considered a safe and minimally invasive alternative marker for pathological evaluation [42]. Evaluation of LV-ECV using CT before surgery for aortic stenosis (AS) has been reported to help predict the risk of postoperative cardiac events [43]. Furthermore, recent studies have shown that CT-based LV-ECV analysis before TAVI predicts postoperative outcome [44].

Furthermore, a certain percentage of patients undergoing TAVI are known to have cardiac amyloidosis, and CT-based LV-ECV evaluation is beneficial in detecting such cases [45]. With recent advances in the treatment of cardiac amyloidosis, timely detection and diagnosis are becoming increasingly important. Current guidelines suggest that delayed contrast-enhanced CT imaging may be useful in detecting cardiac amyloidosis when MRI is not feasible, and its clinical adoption is expected to grow [46].

In cases of atrial fibrillation, tachycardia and irregular ventricular contractions can cause LV remodeling, resulting in decreased LV function [36]. Early evaluation of myocardial injury is warranted, with ablation and pharmacological intervention at the appropriate time to restore LV function.

**Figure 5:**
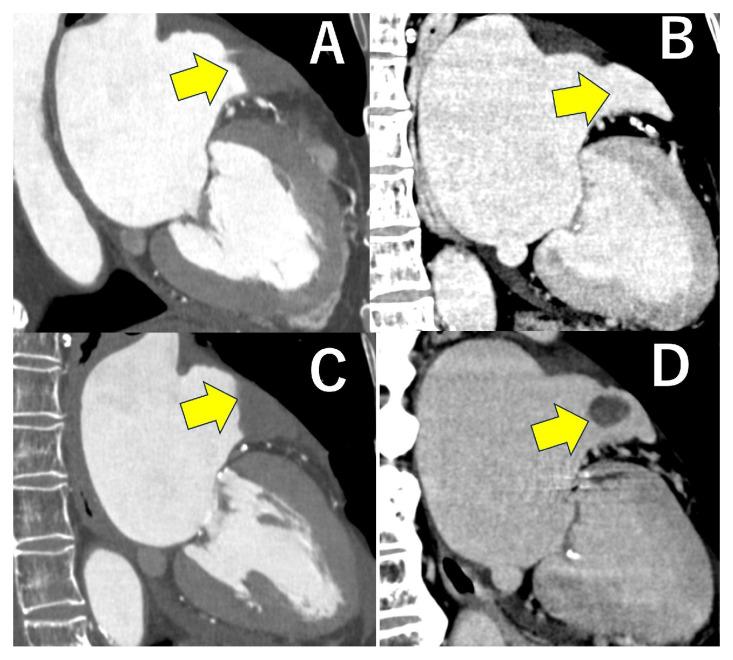
In cardiac computed tomography of patients with atrial fibrillation, delayed contrast agent filling into the left atrial appendage may be observed during the early phase of coronary artery evaluation. In such instances, employing additional late phase contrast images to assess both myocardial injury and left atrial appendage thrombus is highly significant. Upper panel: In the early phase, the contrast agent did not reach the tip of the left atrial appendage, making differentiation from a thrombus difficult (**A**). However, in the late phase, contrast was observed up to the tip, confirming the absence of a thrombus (**B**). Lower panel: Similarly, differentiation was difficult in the early phase (**C**). However, late phase imaging revealed the presence of a thrombus (**D**) (yellow arrows).

Kidoh et al. performed LV-ECV analysis using contrast-enhanced late phase imaging in cardiac CT before catheter ablation for atrial fibrillation (AF). They reported that LV-ECV was significantly higher in persistent AF than in paroxysmal AF [47]. The authors’ group also evaluated LV-ECV on cardiac CT before catheter ablation in patients with AF with reduced LV function and reported significantly lower LV-ECV in the group with LV ejection fraction improved to >50% after the same treatment compared with the group without improvement (31.3 ± 3.3% vs. 35.9 ± 6.6%, *p* < 0.01) [48]. They also reported that scoring using LV-ECV, NT-proBNP, and LV end-diastolic volume was useful for predicting reverse remodeling [49]. Both of these reports may support the importance of intervention from an early stage, when LV myocardial fibrosis is still minimal. Cardiac CT in patients with AF may show delayed contrast arrival in the left atrial appendage during the early contrast phase for coronary artery evaluation. In such cases, evaluating both myocardial damage and left atrial thrombus is meaningful using the additional late contrast phase image (Figure 5).

Furthermore, when considering ventricular resynchronization therapy (CRT), the presence of CRT non-responders, which are estimated to account for about 30% of patients, is often a cause for concern [50]. Although this therapy is indicated when the QRS width of the electrocardiogram (ECG) waveform is 120 ms or more in symptomatic low-functioning heart failure [51]. The search for a method to identify optimal patients for CRT is a current issue.

We conducted a retrospective analysis of LV-ECV in 54 heart failure patients who underwent cardiac CT before CRT implantation. When the CRT responder was defined as a postoperative decrease in LV end-systolic volume of 15% or more by echocardiography [52], LV-ECV was significantly lower in CRT responders (35 ± 6.3% vs. 41 ± 6.8%, *p* = 0.006) [53]. In MRI-based studies, the total scar burden and the presence of scar tissue, especially in the posterolateral wall region commonly used as an LV pacing site, were associated with reduced CRT responsiveness [54,55]. Therefore, myocardial scar tissue was well associated with reduced CRT responsiveness, and LV-ECV on CT reflecting myocardial scar tissue was also considered a good predictor of reduced CRT responsiveness.

We also examined prognosis after CRT implantation. When major adverse events were defined as cardiac death and fatal arrhythmia after CRT implantation, the LV-ECV was significantly higher in the event group (41 ± 7.8% vs. 35 ± 5.0%, *p* = 0.019) [53]. In damaged myocardial tissue, increased extracellular collagen may cause irreversible fibrosis, potentially leading to adverse cardiac events. Therefore, the increase in LV-ECV, which reflects extracellular volume including collagen, was considered a highly sensitive predictor of adverse events.

It should be noted, however, that the image quality of LV late enhancement in CT is positively correlated with the dose of contrast agent and negatively correlated with the patient’s body mass index (BMI). Therefore, image quality may be reduced in patients who require a reduced dose of contrast agent due to renal impairment or in obese patients [2].

## 4. Evaluation of Structural Heart Disease on Computed Tomography

### 4.1. Evaluation of Valvular Heart Disease

The severity of aortic valve stenosis (AS) is generally assessed by echocardiography, but aortic valve (AV) calcium scores on non-contrast CT can be used as an alternative measure when it is difficult to determine whether surgical treatment is necessary based on the results of echocardiography [3].

However, because the leaflets of AV are rapidly moving structures, motion artifacts of them may appear on non-contrast CT images, leading to overestimation of the AV calcification score. Furthermore, data collection is usually restricted to short phases of the cardiac cycle because prospective ECG gated imaging is used to minimize radiation exposure in non-contrast CT. This limitation can result in considerable motion artifacts, especially in patients with high heart rates or arrhythmias.

**Figure 6:**
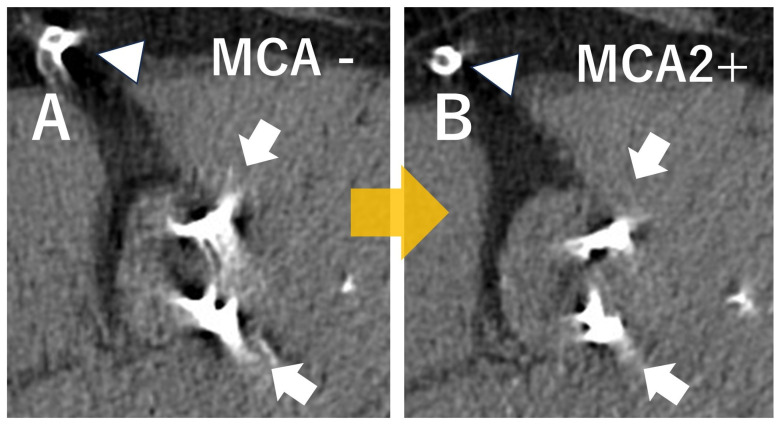
In cases with severe motion artifacts, the second-generation motion correction algorithm (MCA) is useful for aortic valve analysis. Comparison of images of a severely calcified aortic valve (AV) (white arrows) with and without severe motion artifacts is shown. These were calcified AV images from the same patient without the second-generation MCA (SSF2) (**A**) and with SSF2 (**B**). Using the new MCA, the motion artifact of aortic valve calcification and right coronary artery stent are reduced (white arrows and triangles). SSF; Snapshot Freeze.

The latest generation of motion correction algorithms, such as Snapshot Freeze 2 (GE Healthcare, Waukesha, WI, USA), have proven effective in reducing motion artifacts in AV images as well as coronary images. This is especially beneficial in difficult cases [56] (Figure 6).

**Figure 7:**
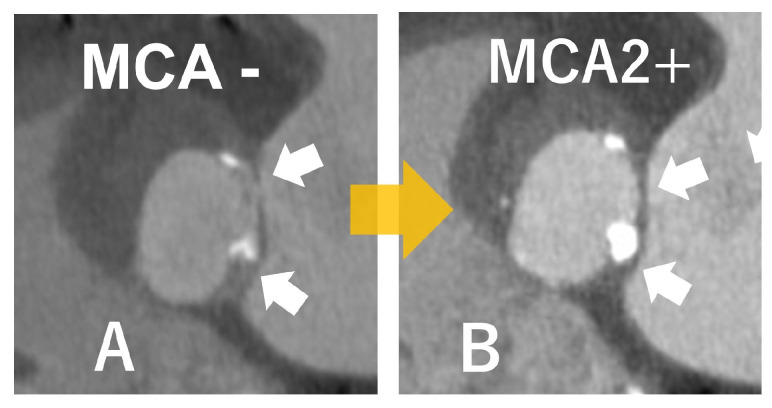
The second-generation motion correction algorithm (MCA) is useful for measuring aortic annulus size in cases with severe motion artifacts. In the case before TAVI, the Aortic annulus size is usually measured on the systolic image at 30% of the R-R wave interval. These are the images of the aortic annulus in the same patient, without the 2nd generation motion correction algorithm (**A**) and with (**B**). The 2nd generation MCA is helpful to decrease the motion artifacts of aortic annulus (white arrows) to accurately measure the size of aortic annulus to decide the artificial valve in TAVI. The new MCA reduces motion artifact of the aortic valve annulus (white arrows). SSF; Snapshot Freeze.

This MCA is also useful for accurately measuring the size of the AV annulus, especially in cases with higher heart rate and severe motion artifact immediately before AV surgery (Figure 7).

### 4.2. The Evaluation of Prosthetic Valve Function

**Figure 8:**
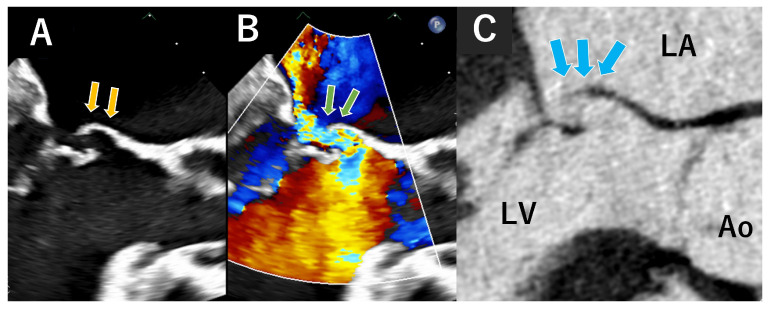
This is a case of infective endocarditis. Transesophageal echocardiography (TEE) revealed the anterior mitral leaflet aneurysm with perforation ((**A**): yellow arrows), which caused severe mitral regurgitation ((**B**): green arrows). Computed tomography showed an aneurysm and perforation of the mitral valve. Valve annulus abscesses were not suspected ((**C**): blue arrows). The mitral valve replacement was performed. LA; left atrium, LV; left ventricle, Ao; aorta.

CT evaluation proves beneficial not only for native valves but also in cases following prosthetic valve implantation. While echocardiography, widely employed for assessing artificial valve function, may sometimes prove challenging for detailed evaluation due to artifacts. Conversely, CT enables four-dimensional observation of the artificial valve through single-heart-cycle imaging, allowing assessment of valve leaflet degeneration, mobility, and abnormal attachments. Therefore, echocardiography is first used to detect valve mobility, increased transvalvular blood flow velocity, or regurgitation. When detailed investigation of the underlying factors is complex using echocardiography alone, evaluation using multiple modalities, including CT, is also recommended [57] (Figure 8).

### 4.3. Evaluation of Congenital Heart Disease on Computed Tomography

**Figure 9:**
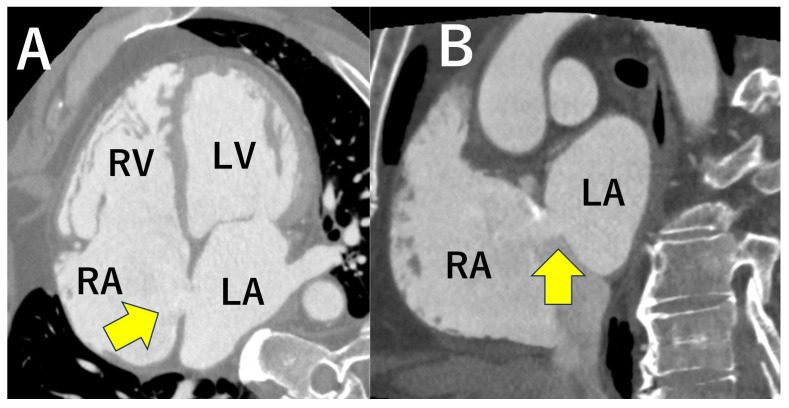
These are atrial septal defects (ASD) on computed tomography ((**A**): four-chamber view, (**B**): short axial view). The ASD secondary defect is well visualized in both images (yellow arrows). LA; Left atrium, RA; Right atrium, LV; Left ventricle, RV; Right ventricle.

CT is useful for three-dimensional evaluation of the heart’s internal structures and not only for evaluating valvular disease but also for identifying congenital abnormalities such as intracardiac shunts (Figure 9) [58].

Especially in young patients with AS, it is essential to evaluate not only valve abnormalities but also complex congenital cardiac malformations. In such cases, CT can be a valuable adjunct to echocardiography. CT is useful in evaluating the aortic valve leaflets and can classify the bicuspid valve [59]. The Sievers classification, which is based on the fusion pattern of the valve leaflets and the presence or absence of a raphe, is the most widely used classification of the bicuspid valve [60]. Type 0 is a true bicuspid valve with no commissure raphe, Type 1 is a bicuspid valve with one commissure raphe, and Type 2 is a bicuspid valve with two commissure raphes (equivalent to a unicuspid valve). Type 1 is further subdivided by the combination of fused valve leaflets [61].

In these patients, ascending aortic enlargement is often observed as a complication [62]. Thus, CT is useful in evaluating aortic anomalies, aortic valves, and coronary artery stenosis, especially in the preoperative setting. Aortic aneurysms are known to be congenital, often associated with a bicuspid aortic valve or subacute aortic valve stenosis. In such severe cases of AS, CT is beneficial for comprehensive evaluation [63].

## 5. Myocardial Functional Analysis on Computed Tomography

In cardiac CT imaging for coronary artery assessment, prospective ECG gating has been employed to minimize radiation exposure by performing short scans during the diastolic or systolic phase, which generally has minimal cardiac motion artifacts [7]. However, the advent of wide-field scanners and other technological advances has enabled further reduction in radiation exposure. Recently, it has become possible to analyze cardiac function by scanning the entire cardiac cycle using CT. Numerous studies employing this protocol have been published [64].

**Figure 10:**
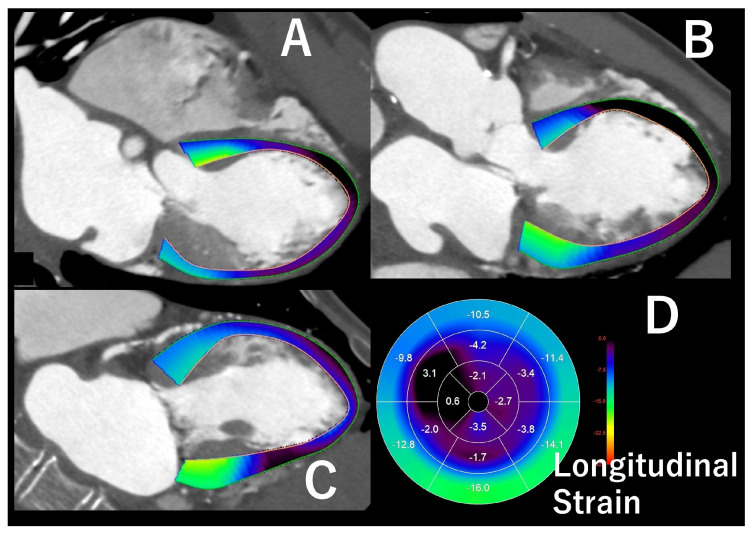
This is a case of takotsubo syndrome. Left ventricular longitudinal strain analysis from cardiac CT performed using a specific software (Ziostation REVORAS v5.3.0.0, Ziosoft Inc., Tokyo, Japan) ((**A**): four-chamber view used for analysis, (**B**): three-chamber view used for analysis, (**C**): two-chamber view used for analysis, (**D**): left ventricular polar map findings from analysis). CT-based LV longitudinal strain analysis revealed reduced strain in a broad area centered on the left ventricular apex.

In particular, cardiac CT performed before TAVI typically requires acquisition of images throughout the cardiac cycle to evaluate the aortic valve complex during systole and the coronary arteries during diastole. Several studies have reported cardiac function assessments using this approach [65]. Recent advances in image analysis software have enabled the measurement of the LV ejection fraction and the analysis of myocardial strain (Figure 10). Echocardiography plays a significant role in strain analysis and has gained attention as a method for analyzing cardiac function, capable of detecting myocardial damage earlier than conventional cardiac function indicators [66]. The advantages of conventional strain analysis by echocardiography include the ability to easily obtain images at the bedside, high temporal resolution, no radiation exposure, and the possibility of repeated evaluations. The advantages of CT include the absence of imaging plane constraints present in echocardiography and reduced variability in acquired images between operators. It may offer significant benefits, particularly in cases where poor echocardiographic image quality is anticipated, such as in obese patients or that post-cardiac surgery. Bernhard B et al. compared myocardial strain analysis using CT and echocardiography in 106 pre-TAVI cases. They reported that the correlation coefficient for global longitudinal strain between the two modalities was very high at 0.816 (*p* < 0.001), whereas those for global circumferential and radial strains were somewhat lower at 0.401 and 0.393 [67]. CT strain analysis, particularly for longitudinal strain, shows good correlation with echocardiographic analysis. Analysis of CT images in candidates for TAVI reported that the group with cardiac adverse events had significantly lower longitudinal strain than the group without such events [68].

## 6. Summary

As described above, the technology of cardiac CT continues to evolve rapidly. It has the potential to play an equal or even greater role in overcoming the weaknesses of other modalities. Cardiac CT, through its technological evolution, has expanded beyond traditional coronary artery assessment to encompass a wide range of evaluation targets, including myocardial tissue, valves, and shunt lesions (Table 2). The combination of these new technologies is expected to significantly reduce radiation exposure, potentially making cardiac CT for screening purposes a realistic option in the future. With the addition of new AI technologies, advancements in AI-driven automated diagnosis and risk stratification, as well as integrated analysis with other imaging diagnostics, are also anticipated to progress.

As cardiac CT continues to expand its field of application in the treatment of heart failure and arrhythmias, clinicians need to understand cardiac CT examinations better and update their knowledge of the latest findings.

## Figures and Tables

**Table 1:** New technologies and main utility of them on cardiac CT.

New Technology	Main Utility of New Technology
Multislice detector	Evaluation of coronary artery lumen and plaques, myocardium, heart valve; Reduction in radiation
High speed gantry rotation	Evaluation of coronary artery lumen and plaques
Motion correction algorism	Evaluation of coronary artery lumen and plaques, aortic annulus, heart valve
Deep learning reconstruction	Evaluation of myocardium; Reduction in radiation
Dual energy CT	Evaluation of coronary artery lumen and plaques, myocardium
Photon counting CT	Evaluation of coronary artery lumen and plaques, myocardium; Reduction in radiation
Ultra high-resolution CT	Evaluation of coronary artery lumen, plaques and stents

**Table 2:** Target lesions of evaluation of cardiac CT.

Targets of Evaluation of CT	What Should Be Evaluated on CT
Coronary artery	stenosis, plaque, calcification, fractional flow reserve
Myocardium	late enhancement (fibrosis), myocardial fat, motion (strain)
Valve	motion, calcification, annulus, vegetation
Shunt disease	shunt flow, defect
Mass	thrombus, tumor

## Data Availability

No data related to this study will be shared.
